# Contrasting Invasion Strategies, Convergent Outcomes: Establishment of *Zaprionus tuberculatus* and *Ceroplastes ceriferus* in Italy

**DOI:** 10.3390/insects17020198

**Published:** 2026-02-12

**Authors:** Francesco Nugnes, Carmela Carbone, Fortuna Miele, Feliciana Pica, Sara Pierro, Raffaele Sasso, Mariagrazia Bodini, Umberto Bernardo

**Affiliations:** 1 Institute for Sustainable Plant Protection, National Research Council (IPSP-CNR), P.le E. Fermi, 1, 80055 Portici (NA), Italy; francesco.nugnes@cnr.it (F.N.); carmelacarbone@cnr.it (C.C.); fortunamiele@cnr.it (F.M.); sarapierro@cnr.it (S.P.); mariagraziabodini@cnr.it (M.B.); umberto.bernardo@cnr.it (U.B.); 2 ENEA CR Casaccia, SSPT-AGROS-AGRI4.0, Via Anguillarese, 301, 00123 Rome, Italy; raffaele.sasso@enea.it

**Keywords:** biological invasions, establishment, founder effects, genetic paradox, host plants, mitochondrial haplotypes

## Abstract

Global warming and international trade are accelerating biological invasions, making the Mediterranean basin a hotspot for alien arthropods. Two invasive species, the drosophilid *Zaprionus tuberculatus* and the wax scale *Ceroplastes ceriferus*, were recently detected in central-southern Italy. Surveys conducted between 2023 and 2024 detected *Z. tuberculatus* across several sites in Campania and Lazio, and *C. ceriferus* in Campania. *Zaprionus tuberculatus* was reared from ten host plants, including three new records, and reached its highest abundance on persimmon. Laboratory assays showed that *Z. tuberculatus* adults reproduced only on intact fig fruits and blueberry, indicating a limited risk for most crops. *Ceroplastes ceriferus* was found on four hosts, confirming its polyphagy and establishment in southern Italy. Molecular analyses revealed two COI haplotypes in the Italian populations of both species, while three haplotypes were detected globally in *Z. tuberculatus* and seven in *C. ceriferus*. Sequence divergences were moderate, indicating limited but appreciable mitochondrial differentiation among geographic populations. In *Z. tuberculatus*, the COII haplotype detected in Italy did not match available African sequences, underscoring gaps in reference data and complicating the reconstruction of invasion routes. Despite contrasting ecological traits and dispersal strategies, both species achieved successful establishment in Italy, showing that ecological plasticity and human-mediated transport can offset reduced genetic diversity. These records expand the known European range of both species and highlight the need for coordinated surveillance, genetic monitoring, and preventive measures to limit further introductions in the Mediterranean region.

## Simple Summary

Two non-native insect species, the fruit fly *Zaprionus tuberculatus* and the wax scale *Ceroplastes ceriferus*, have recently extended their distribution range into Italy. Although they differ greatly in behaviour and biology, the fruit fly being highly mobile and the wax scale remaining fixed on the plant, both established themselves in local environments through different invasion pathways. This study examined where these species occur, which plants they attack, and whether they are able to reproduce under Italian conditions. The insects were also analysed genetically to understand how much variation they possess and how Italian populations relate to those from other parts of the world. Such information helps clarify how these species arrive, spread, and adapt to new areas. The findings show that both insects are already established on several host plants and can persist under Mediterranean climate conditions. Despite carrying only limited genetic variation, both species successfully settled in the region. This outcome reflects what is known as the “genetic paradox”, in which invasive species can thrive even with reduced genetic diversity, highlighting the need for continued surveillance and a better understanding of the mechanisms that enable successful establishment.

## 1. Introduction

In recent decades, rising global temperatures have expanded the ecologically suitable range for invasive pests, increasing the vulnerability of temperate regions and driving shifts in pest dynamics [1,2,3]. According to the Intergovernmental Panel on Climate Change [4], the climate will further alter ecosystems, creating conditions favourable for the establishment of tropical and subtropical species in previously unsuitable areas [5].

The Mediterranean basin has been recognised as a hotspot for alien insects due to its mild climate and ecological heterogeneity [6]. Countries like France, Italy, and Spain, which combine Mediterranean, continental, and mountainous environments, offer ideal conditions for the establishment of alien species [7]. Italy and France are most prominently regarded as primary entry hubs for early detection points in Europe [8].

Despite strengthened phytosanitary measures, the number of unintentionally introduced pests, especially insects and mites, continues to rise in Italy [9,10,11]. Arthropods, and particularly insects, account for more than 90% of alien invertebrates established in Europe [12,13], a predominance explained by their high species diversity, small size, rapid life cycles, flexible feeding habits, and close association with human activities [14,15].

In addition to climate change, global trade and human mobility are major drivers of invasions, posing threats to ecosystems, agriculture, and local economies [16,17,18,19]. The growing international trade in tropical fruits, such as avocado, banana, cherimoya, guava and mango, is a frequent introduction pathway. It occurs both through official commercial routes and personal luggage [20,21].

The ecological and economic consequences of biological invasions are substantial. They generate major costs worldwide, from direct crop losses to long-term management [22,23].

In Europe, damages have been estimated in the order of hundreds of billions of euros, with agriculture among the most affected sectors [24,25]. In Italy, the management of pests such as *Bactrocera dorsalis* Hendel (Diptera: Tephritidae) and *Popillia japonica* Newman (Coleoptera: Scarabaeidae) already requires considerable resources, and costs are expected to increase without timely interventions [26,27,28].

To address these challenges, the European Union adopted Regulation No. 1143/2014, aimed at preventing and managing the introduction and spread of invasive alien species to protect biodiversity and ecosystem services [29]. Key elements include a solid understanding of invasion processes, strengthening biosecurity at entry points [30,31] and the implementation of rapid responses in the early phases of invasion, which are among the most cost-effective strategies to reduce long-term impacts [32,33,34,35].

Within this context, the first detections in Campania of two invasive species already recorded in Italy are reported: *Zaprionus tuberculatus* (Malloch) (Diptera: Drosophilidae), also detected in Lazio during this study, and *Ceroplastes ceriferus* (Fabricius) (Hemiptera: Coccidae).

*Zaprionus tuberculatus*, the vinegar fly, an Afro-tropical drosophilid first described in South Africa [36], has since achieved a broad global distribution, with records across Europe, Asia, and the Americas [37,38,39,40,41]. It is highly polyphagous, exploiting a wide range of host fruits, particularly overripe, damaged, or fallen fruits, but also capable of developing on intact fruits under certain conditions; its short life cycle and high reproductive potential facilitate rapid population growth and colonisation of new habitats [41,42]. Despite its expansion, genetic information on this species remains limited, and the effectiveness of DNA barcoding has been questioned [36].

*Ceroplastes ceriferus*, the Indian wax scale, is a polyphagous species native to Asia and reported in Europe since 2001 [43,44]. It infests hosts from over 60 plant families [45,46] and has been listed as a dangerous invasive pest in Italy since 1988 [47]. Its thick wax test protects it from natural enemies and control measures, and its host range and adaptability make it a serious threat to agriculture and ornamental plants [48].

Although the two species differ markedly in their life-history strategies, *Z. tuberculatus* is a mobile, multivoltine drosophilid, whereas *C. ceriferus* is a sedentary, univoltine scale insect. Despite these differences, both represent contrasting invasion pathways that have nonetheless resulted in successful establishment.

Recent theoretical frameworks indicate that many invasive species can successfully establish despite demographic bottlenecks and reduced mitochondrial diversity, a phenomenon known as the “genetic paradox” of biological invasions [49]. Most empirical assessments of this paradox have focused on single-species case studies, leaving unresolved the question of whether invasive success under reduced genetic variability is contingent on specific life-history traits or represents a more general outcome across divergent invasion strategies. In this context, these two co-occurring invaders provide a useful system to explore how contrasting ecological strategies interact with genetic variability during establishment.

The aims of this study were to:

(1) Characterise *Z. tuberculatus* and *C. ceriferus* distributions and host associations;
(2) Generate novel genetic data for *Z. tuberculatus*, supporting taxonomic and phylogeographic studies, and experimentally assess its ability to infest healthy fruits of different host species;
(3) Evaluate evidence of acclimatization of both species to Italian environmental conditions;
(4) Compare levels of genetic variability in Italian populations with those reported worldwide;
(5) Assess whether contrasting life-history strategies are associated with different patterns of genetic variability during invasion and evaluate whether successful establishment under reduced genetic diversity supports the genetic paradox across divergent invasion strategies.

## 2. Materials and Methods

### 2.1. Sampling Activity

Between 2023 and 2024, monitoring activities conducted in Campania and Lazio detected the presence of *Z. tuberculatus* in both regions, and *C. ceriferus* in Campania only, in areas where surveillance efforts were primarily focused on the outbreak of *B. dorsalis*. These records broadened the scope of the survey and allowed for the targeted data collection on both species. Sampling was conducted in mixed orchards and private gardens, with a high degree of host plant heterogeneity, as well as greenhouses, garden centres, and border control points, in accordance with Regulation (EU) 2019/2072 “https://eur-lex.europa.eu/eli/reg_impl/2019/2072/oj/eng” (accessed on 30 October 2025).

#### 2.1.1. *Zaprionus tuberculatus*

To monitor fruit flies, twenty fruits were collected from the ground and another twenty directly from trees in each surveyed area where fruits showed symptoms attributable to Tephritidae infestation.

Infested fruits were processed following the protocol described in [50] to allow larval development and pupation, as well as to evaluate the potential presence of natural enemies. Emerging adult specimens were preserved in 90% ethanol (Carlo Erba Reagents S.r.l., Milan, Italy), stored individually in Eppendorf tubes, and catalogued with the collection date, location, and host plant species.

As part of the Regional Action Plan for *B. dorsalis* experimental “attract and kill” traps were deployed. These red traps Decis Trap^®^ (Bayer CropScience AG, Monheim am Rhein, Germany) had the inner lid treated with Deltamethrin and were baited with methyl-eugenol (GEA s.r.l.; Milan, Italy). The traps, which are routinely sold pre-baited with ethyl acetate, 2,6-di-tert-butyl-4-methylphenol, acetoin, and 3-(methylthio) propanol, were thoroughly washed before the trial with a solution of distilled water and commercial bleach (ACE, Fater S.p.A., Pescara, Italy) (10:1, *v*/*v*).

#### 2.1.2. *Ceroplastes ceriferus*

To monitor scale insects, visual examinations were primarily performed on secondary branches and the underside of leaves. Sampling was carried out across ten sites. Adult specimens were collected directly from host plants using entomological spatulas and placed in Falcon tubes or Petri dishes. All samples were transported to the CNR-IPSP laboratory in sealed bags and kept in isolation in a climatic chamber at 25 °C and 70% RH to detect the eventual emergence of associated parasitoids.

### 2.2. Evaluation of Capacity to Damage Fruits

These assays were designed as qualitative tests to assess whether oviposition and development could occur on intact fruits, rather than to quantify host preference or reproductive performance. Adult flies emerging from infested field-collected fruits were reared in Bug Dorms cages (D30 × W30 × H30 cm) following the method described by [51]. Groups of ten adults (five males and five females) were simultaneously exposed to the fruits for 72 h. All exposure assays were conducted under controlled conditions (25 ± 1 °C, 70 ± 10% RH, [12:12 photoperiod]). For *Diospyros kaki* Thunb. (persimmon), two intact, untreated ripe fruits were used per replicate, whereas for *Vaccinium corymbosum* (blueberry), *Vitis vinifera* L. (grape), *Fragaria × ananassa* (Duchesne ex Weston) (strawberry), *Ribes rubrum* L. (red currant), and *Ficus carica* L. (fig), each replicate consisted of approximately 125 g (blueberries, red currants) and 250 g (grapes, strawberries, figs) of healthy, undamaged fruits.

The test was replicated three times for each fruit species. Fruits were placed directly inside the cages and were not replaced during the exposure period. After exposure, fruits were examined daily for signs of oviposition or larval activity for at least 15 days.

The integrity of the fruits was carefully checked under a microscope (40× magnification) before and after exposure to exclude any pre-existing wounds or micro fissures that could facilitate oviposition. This procedure ensured that the assay reproduced natural infestation only on healthy fruits. The use of intact fruits followed previous observations that *Z. tuberculatus* can develop in several host species under different experimental conditions [52].

### 2.3. Integrative Identification

#### 2.3.1. Morphological Identification

Adults, either collected directly from traps or host plants or collected when they emerged from sampled fruits, were identified based on morphology using existing taxonomic keys. All specimens were examined under a Leica M165C auto-montage microscope (Leica Microsystems, Mannheim, Germany) equipped with a Leica DFC450 digital camera to obtain multifocal images.

Adult drosophilid specimens were identified using the keys of [53,54]. For detailed morphological analysis, nine Drosophilidae specimens were slide-mounted in Euparal and examined under a Zeiss Axiophot 2 microscope (Carl Zeiss, Oberkochen, Germany), following the methodology of [36].

Scale specimens were identified according to the methodology described by [55,56]. Identification was further supported using the key for *Ceroplastes* species occurring in Europe provided by [57].

#### 2.3.2. Molecular Analysis

A total of 40 samples, including 21 adults of *Z. tuberculatus* and 19 of *C. ceriferus*, were selected for molecular analysis. Selection criteria included collection sites (with at least one specimen per site), distance between sites (priority given to the most distant), and host species (at least one specimen per host) (Table 1 and Table 2).

DNA was extracted using the Chelex-Proteinase K protocol described by [58], PCR amplicons were verified on a 1.2% agarose gel electrophoresis and subsequently sequenced. To assess genetic variability, mitochondrial markers COI and COII were selected due to their extensive coverage in public genetic databases (Genbank and BOLD) for the target taxa, allowing direct comparison between the newly generated sequences and previously published data.

##### *Zaprionus* *tuberculatus*

DNA was extracted from the right hind leg, and fragments of the mitochondrial COI and COII genes were amplified. COI amplification was performed as described in [59]. COII amplification was carried out using the specific primers TL2-J-3037 and TK-N-3785, following the PCR protocol reported by [36]. The COII region was also targeted because homologous sequences from African populations are available in GenBank, allowing to explore possible insights into the geographic origin of the Italian specimens [36].

##### *Ceroplastes* *ceriferus*

Whole individuals were washed with distilled water and carefully cleaned of wax using a fine needle prior to DNA extraction. The entire body was used for DNA extraction. COI gene amplification was performed with the primers and thermocycler condition described by [60], which ensure effective amplification of the target fragment.

### 2.4. Evaluation of Genetic Diversity

#### *Zaprionus tuberculatus* and *Ceroplastes ceriferus*

The obtained sequences were assembled and manually edited using BioEdit v.7.0 [61]. The presence of potential stop codons or pseudogene was verified with EMBOSS Transeq “https://www.ebi.ac.uk/jdispatcher/st/emboss_transeq” (accessed on 5 October 2025)”. Final sequences were compared against the GenBank “https://www.ncbi.nlm.nih.gov/genbank/” (accessed on 5 October 2025)” and BOLD databases “https://portal.boldsystems.org” (accessed on 30 October 2025). The sequences were subsequently submitted, and the corresponding accession numbers are provided in Table 1 and Table 2.

Genetic distances based on the COI gene for *Z. tuberculatus* and *C. ceriferus*, and the COII gene for *Z. tuberculatus*, were estimated using MEGA v.11 with the uncorrected p-distance model [62]. Intra-specific relationships among the collected samples, as well as additional *Z. tuberculatus* and *C. ceriferus* specimens from worldwide locations, were also examined. All available homologous COI and COII sequences for these taxa, retrieved from GenBank and BOLD (accessed on 5 April 2025), were aligned with the dataset obtained in this study for subsequent analyses.

Relationships among haplotypes of both species were inferred using a statistical parsimony approach implemented in TCS v.1.21 based on COI and, for *Z. tuberculatus*, also on COII. The resulting networks were examined and visualised with tcsBU, a web-based application that extends the original functionality of the TCS [63].

## 3. Results

### 3.1. Sampling Activity

#### 3.1.1. *Zaprionus tuberculatus*

**Figure 1:**
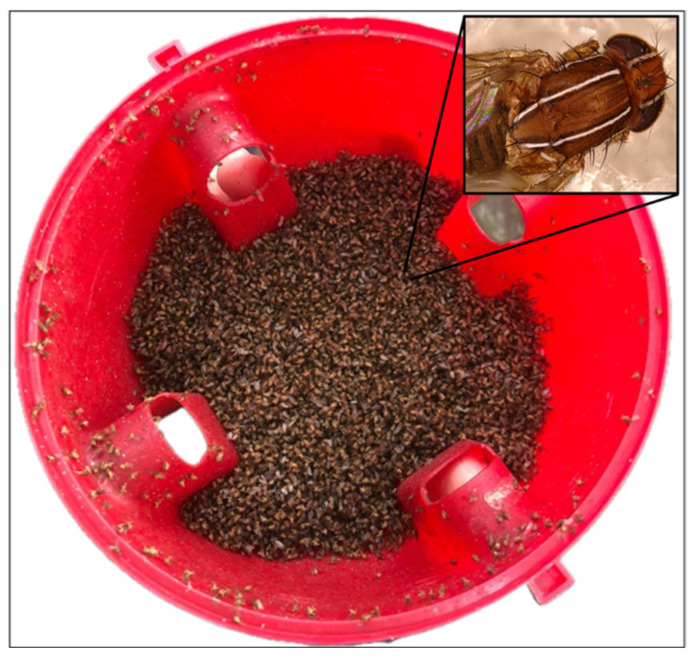
Numerous specimens of *Zaprionus tuberculatus* captured in an experimental “attract and kill” Decis trap (Bayer Crop Science) adapted for the control of *Bactrocera dorsalis*.

Adults of *Z. tuberculatus* emerged from infested fruits are listed in Table 1. More than 400 fruits were collected and examined, and *Z. tuberculatus* adults emerged from ten different host species (Table 1). In total, 179 adults were obtained, representing the first record of this species in the entomofauna of Campania and Lazio. In addition, many specimens were captured in experimental “attract and kill” Decis-trap (Bayer Crop Science), which had been adapted for use against *B. dorsalis*. These traps, baited with methyl-eugenol and fitted with a lid treated with deltamethrin, also proved effective in attracting Drosophilidae species (Figure 1). Captures in the Decis-trap were higher than in standard methyl-eugenol traps, suggesting that residual attractants from the original formulation enhanced its performance.

**Figure 2:**
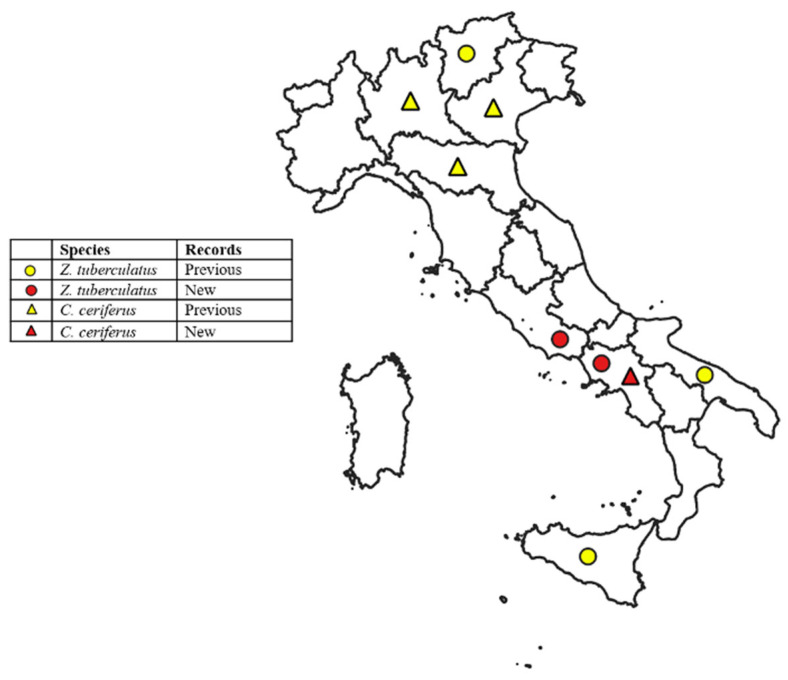
Distribution of *Zaprionus tuberculatus* and *Ceroplastes ceriferus* in Italy, including new and previous records.

The distribution of positive sampling sites is shown in Figure 2.

#### 3.1.2. *Ceroplastes ceriferus*

A total of 65 adult specimens of *C. ceriferus* were collected from four different host plants (Table 2).

The geographical distribution of positive sampling sites in Campania is shown in Figure 2.

From the examined samples of both *Z. tuberculatus* and *C. ceriferus*, no parasitoid emergence was observed.

### 3.2. Evaluation of Capacity to Damage Fruits

Healthy persimmon, strawberry, red currant, and grapes fruits exposed to *Z. tuberculatus* showed no signs of oviposition, egg presence, or larval development. In contrast, oviposition occurred on healthy fig fruits and on blueberries, from which adults successfully emerged.

### 3.3. Integrative Identification

#### 3.3.1. Morphological Identification

All specimens were morphologically examined according to the available taxonomic literature. Adults of *Z. tuberculatus* displayed the diagnostic characters reported in existing keys, including the distinctive longitudinal body stripes and male genitalia features. Adult females of *C. ceriferus* matched the published descriptions of the species, showing the characteristic thick wax covering and typical body shape.

#### 3.3.2. Molecular Analysis

##### *Zaprionus* *tuberculatus*

**Figure 3:**
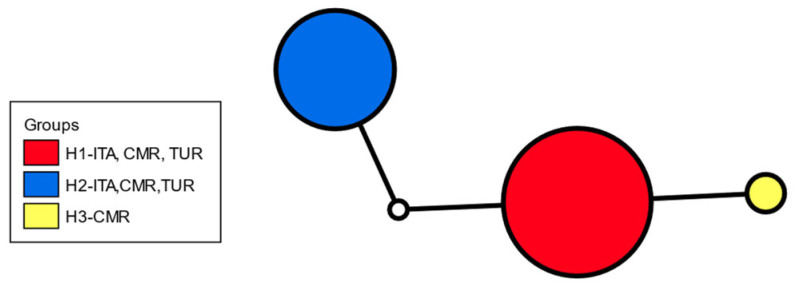
COI haplotype network of *Zaprionus tuberculatus*. Circle size is proportional to the number of individuals; colours indicate haplotypes (ITA, Italy; CMR, Cameroon; TUR, Turkey.

Worldwide, three COI haplotypes were detected: two shared with the Italian population (H1, H2) and one (H3) found exclusively in Cameroon that differed by a single synonymous nucleotide substitution. Within the Italian samples, 24 COI sequences clustered into two haplotypes (11 in H1 and 13 in H2), which differed from each other by two base pairs (Table 1, Figure 3, Table S1). 

**Figure 4:**
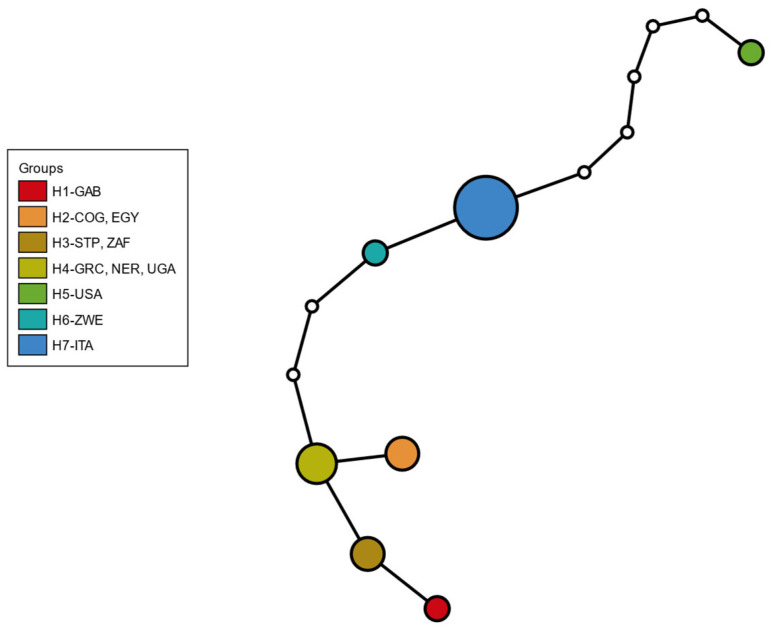
COII haplotype network of *Zaprionus tuberculatus*. Circle size is proportional to the number of individuals; colours indicate haplotypes (COG: Republic of the Congo; EGY: Egypt; GAB: Gabon; GRC: Greece; ITA: Italy; NER: Niger; STP: São Tomé and Príncipe; UGA: Uganda; USA: United States of America; ZAF: South Africa; ZWE: Zimbabwe).

For the COII gene, all eight Italian specimens showed the same haplotype. After alignment with worldwide sequences (18 in total), seven distinct haplotypes were identified, and the Italian haplotype (H7) had never been reported previously (Table 1, Figure 4, Table S2).

##### *Ceroplastes* *ceriferus*

**Figure 5:**
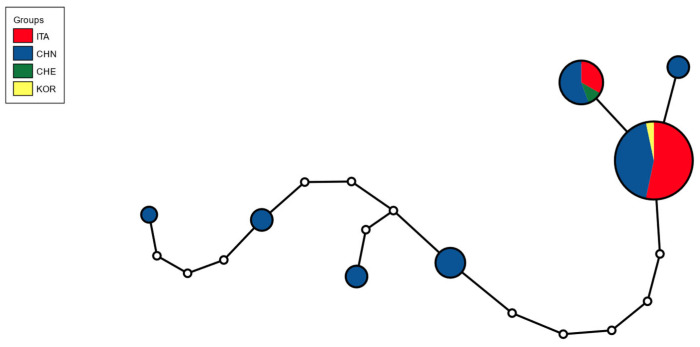
COI haplotype network of *Ceroplastes ceriferus*. Circle size is proportional to the number of individuals; colours indicate haplotypes (CHE: Switzerland; CHN: China, ITA: Italy, KOR: South Korea).

Worldwide, seven COI haplotypes were detected after integrating 50 sequences from public databases (Table 2, Figure 5, Table S3). Two of these haplotypes (H1 and H2) occurred in the Italian specimens, which differed by one nucleotide.

### 3.4. Evaluation of Genetic Diversity

#### 3.4.1. *Zaprionus tuberculatus*

The mean intra-group genetic distance for the COI portion was 0.13% (±0.103%) (Table 3).

Genetic distances among the analysed groups were generally low. The highest distance was observed between specimens from Cameroon (CMR) and Italy (ITA) (0.20%). In almost all cases, inter-group differences corresponded to a single-nucleotide substitution, highlighting a substantial genetic affinity among the analysed samples.

Furthermore, the COII obtained sequences differed from all those from other countries available in the databases. Intra-group genetic distance was observed only in the African (AFR) samples (0.26%) (Table 3). The greatest divergence among inter-group distances (Table 3), was observed between the African and American (USA) groups (1.5%), whereas the lowest was between the American and Italian groups (0.63%).

#### 3.4.2. *Ceroplastes ceriferus*

The mean intra-group genetic distance was 0.40% (±0.134% SE) (Table 4), with the lowest values observed in the Italian samples (ITA). All analysed populations showed very limited genetic divergence. Two haplotypes were detected in Italy, both shared with populations from China, one also recorded in South Korea and the other in Switzerland. The mean inter-group genetic distance was 0.32% (±0.146% SE). The Chinese samples displayed the highest haplotype diversity, whereas other populations were represented by one or two haplotypes identical to those found in China.

## 4. Discussion

### 4.1. Zaprionus tuberculatus

The results confirm the establishment of *Z. tuberculatus* in Campania and Lazio, expanding its known distribution in Italy (Figure 2). Previous records include sporadic detections in Apulia and the first report from the European mainland in Trentino-Alto Adige (2013). Since then, the species has rapidly spread across Europe, with established populations in mainland France and additional records from Sicily and Corsica, where it was collected in citrus orchards and ornamental plants [64]. It has also been reported from North Africa [37,39,65,66]. Consistent with this observed expansion, species distribution models based on bioclimatic variables predict that *Z. tuberculatus* could further expand its distribution within Europe and across the American continent under climatic conditions suitable for its development [41].

In the present surveys, the species was reared from ten host plants, including three new records (*Eriobotrya japonica*, *Juglans regia*, *Ziziphus jujuba*) (Table 1). Among all hosts, the high number of individuals emerging from *D. kaki* likely reflects the temporal overlap between fruit ripening (September–October) and population peaks, together with the increased vulnerability of overripe fruits, whose skin becomes particularly delicate.

In mixed orchards, the staggered ripening of different fruit species provides a continuous supply of suitable substrates during autumn, promoting the build-up and persistence of *Z. tuberculatus* populations. Despite abundant captures, the species has not yet been consistently recorded during late winter–spring, unlike endemic tephritids such as *C. capitata*. This seasonal gap suggests that *Z. tuberculatus* survives the winter only with very low success, leading to a demographic bottleneck and scarce spring populations, as also observed in *Zaprionus indianus* Gupta (Diptera: Drosophilidae) [67]. Taken together, these observations are consistent with a multivoltine life cycle for *Z. tuberculatus* under Mediterranean conditions, although the effective number of generations per year is likely constrained by winter mortality. Laboratory trials excluded the oviposition and development on unwounded persimmon, strawberry, red currant, and grapes, confirming the behaviour of *Z. tuberculatus* as a secondary coloniser of damaged or overripe fruits.

In contrast, oviposition and successful progeny were consistently obtained on healthy fig fruits, in agreement with field observations where numerous figs showed multiple egg depositions around the ostiole. This confirms figs as the only intact fruit that regularly supports development and a key resource for the establishment and seasonal increase in *Z. tuberculatus* in Mediterranean environments.

A limited number of adults also emerged from blueberries, indicating that the species can complete development on this fruit, although only marginally. Blueberry, therefore, appears to function as an occasional host of low suitability, with limited relevance for population maintenance compared with figs.

The strong association with ripe figs suggests that establishment in Mediterranean areas is facilitated by the widespread availability of this fruit during late summer. The dependence on access points to the pulp, rather than on fruit chemistry, may further explain the reliance of the species on figs, while reducing the potential risk for most commercial crops. El-Sabrout et al. [68] showed that *Z. tuberculatus* can be reared for over 60 generations under controlled conditions, confirming its high reproductive capacity. Females from Egyptian populations survived desiccation better at lower temperatures than sub-Saharan lineages, suggesting adaptation to temperate climates. The species was also more frequent in fruit-rich agricultural areas, unlike *Z. indianus*, which dominated urban sites.

In regions where *C. capitata*, *Drosophila suzukii* (Matsumura) (Diptera: Drosophilidae) and other tephritids are abundant, such as the Mediterranean basin, potential interactions with *Z. tuberculatus* should also be considered. *Ceratitis capitata* is long established in the region, whereas *B. dorsalis*, recently detected in Campania, showed activity periods that overlap with those of *Z. tuberculatus* during late summer and autumn [69]. *Drosophila suzukii* is also active in the same period in many Mediterranean areas [70], adding a further potential source of fruit wounds that may facilitate secondary colonisation by *Z. tuberculatus*. Such temporal and trophic overlap may influence the population dynamics of all species involved and potentially amplify fruit damage, although specific studies under Mediterranean conditions are still lacking.

Similar laboratory assays conducted in Turkey reported successful development on additional hosts, including strawberries, pears, and pomegranates [52]. Differences among studies may reflect variability in fruit integrity, cultivar susceptibility or environmental conditions, emphasising the need for standardised experimental protocols. Comparable risks were noted in Tunisia [65] and France [66]. The potential impact of this species was also recognised by the European and Mediterranean Plant Protection Organization, which placed *Z. tuberculatus* on its Alert List from 2016 to 2020, citing risks for figs and other soft fruits [71]. The present results also suggest that Decis-traps may provide an effective tool for monitoring *Z. tuberculatus* and for mass trapping. The invasion dynamics observed in Italy resemble those of the congeneric species *Z. indianus* in Brazil, where the species rapidly spread and became dominant under subtropical conditions [37,72,73].

The limited genetic variability observed in Italian populations, as indicated by the presence of only two COI haplotypes differing by two base pairs and identical COII sequences, likely reflects a founder effect caused by a recent introduction involving a restricted number of individuals. The presence of a COII haplotype that does not match any known African sequences, although Africa is considered the native range of the species [36], prevents the identification of the precise origin of the Italian population.

This mismatch likely reflects the limited geographic coverage of available reference data, as most COII sequences in public databases originate from African populations.

The absence of this haplotype in Africa could therefore be due either to unsampled source populations or to indirect introduction routes through other regions where COII has not yet been surveyed. Similar situations have been reported for other invasive insects, where incomplete or geographically biased datasets hampered the reconstruction of invasion pathways [74].

### 4.2. Ceroplastes ceriferus

This study provides the first official record of *C. ceriferus* in Campania, documenting its establishment in southern Italy. The history of introductions in Europe shows that *Ceroplastes* species have been repeatedly recorded in the Mediterranean. In Italy, *Ceroplastes japonicus* (Gray) (Hemiptera: Coccidae) was reported in 1984 and *C. ceriferus* in 2001 and both species later spread to several European countries [75]. This evidence supports the view that southern Europe represents a permanent entry point for invasive wax scales. The species was detected on four hosts (*D. kaki*, *Laurus nobilis*, *Morus* sp., *Photinia serratifolia*), confirming its polyphagy and ecological flexibility.

Morphological identification was supported by molecular data, which revealed two COI haplotypes differing by only 1 base. The two haplotypes detected in Italy were identical to haplotypes previously reported from China, with one also occurring in Switzerland and the other in South Korea. The temporal sequence of detections in Europe suggests the possibility of a “bridgehead effect,” in which Italy acted as a secondary source for the subsequent introduction of the species into Switzerland [76,77]. This scenario supports the hypothesis of human-mediated dispersal of a limited Asian genetic lineage through plant trade and ornamental exchanges, consistent with the species being native to Asia [78].

The within-group variability of the Chinese samples exceeded the genetic distances observed among populations from different countries, which is consistent with higher heterogeneity in the native range.

The occurrence of two haplotypes suggests that the introduced population is genetically heterogeneous, albeit to a limited extent. Comparative studies conducted in China revealed low intraspecific divergence within the genus *Ceroplastes*, with clear species-level separation but close clustering of *C. ceriferus* and *Ceroplastes pseudoceriferus* Green (Hemiptera: Coccidae) [60].

The trade of ornamental plants has long represented a major pathway for the introduction and spread of exotic scale insects in Italy [48]. Most *Ceroplastes* species in the Palaearctic are non-native, with a circum-Mediterranean distribution and repeated interceptions in central and northern Europe [75]. This pathway is highly relevant for *C. ceriferus*, frequently intercepted on ornamental plants, especially *Ficus microcarpa* L.f. [21], before its establishment. Recent records from Switzerland and Turkey extend the known distribution of the species and confirm its presence in different parts of the Mediterranean region [76,79].

The present findings provide further evidence of the shift from repeated interceptions to local establishment in southern Italy, where the species was observed during winter and the following spring, confirming its acclimatization. Infestations were limited, affecting only a few plants and restricted branches, but their persistence across seasons indicates that *C. ceriferus* has already established viable populations. The occurrence on both fruit and ornamental hosts highlights its ability to move between urban and agricultural contexts. This ecological flexibility, combined with confirmed overwintering, suggests that even small founder populations can represent a latent risk for further spread.

### 4.3. Genetic Variability and Invasion Success

The establishment of *Z. tuberculatus* and *C. ceriferus* in Italy, despite their extremely reduced mitochondrial variability, contributes to the long-standing paradox in invasion biology [49,80]. Although they display opposite ecological strategies, with *Z. tuberculatus* relying on active dispersal and host switching, and *C. ceriferus* spreading passively through the trade of infested plants, both species show comparable genetic patterns, characterised by few mitochondrial haplotypes and limited divergence.

Recent syntheses and genomic studies indicate that this so-called genetic paradox is often only apparent. Indeed, successful establishment may occur despite extremely reduced mitochondrial diversity, with adaptive responses frequently relying on standing nuclear variation or key functional loci rather than mitochondrial markers [77,81,82].

This convergence suggests that successful establishment can arise through different ecological pathways when suitable host availability and human-mediated dispersal are present. The two species also represent different stages of invasion: *Z. tuberculatus* is a recent introduction that has expanded rapidly across southern Europe, whereas *C. ceriferus*, intercepted decades ago, is now confirmed as established.

Although bottlenecks, founder effects and genetic drift reduce diversity and adaptive potential, many invaders reach high abundance, as observed here. Only two highly similar haplotypes were detected in both species, yet establishment was not hindered. Several mechanisms may explain this paradox: bottlenecks may purge deleterious alleles [83], favour social or ecological traits [84], or preserve additive variance [85].

The contrasting biology of the two pests underscores that invasion success is not determined by dispersal mode alone. *Zaprionus tuberculatus* is a mobile drosophilid able to switch hosts, whereas *C. ceriferus* is a sessile scale insect spread through plant trade.

Their convergence toward the same invasion outcome, successful establishment despite low genetic variability, highlights the role of ecological adaptability and introduction dynamics.

Comparable patterns are known in other invasive insects: *Phthorimaea absoluta* (syn. Tuta absoluta), *Leptocybe invasa* Fisher and La Salle (Hymenoptera, Eulophidae), *Coptodisca lucifluella* (Clemens) (Lepidoptera, Heliozelidae), and *Cameraria ohridella* Deschka and Dimić (Lepidoptera, Gracillariidae), all spread in Europe with very low mitochondrial diversity [86,87,88,89,90]. These cases show that introductions often involve 1–2 haplotypes, yet invasions can still succeed.

Genome-wide approaches indicate that adaptation may rely on key nuclear genes related to host use, physiology, and stress tolerance [15,82], even when mitochondrial variability is minimal. Together, these two case studies illustrate how distinct invasion strategies, active dispersal and ecological opportunism in *Z. tuberculatus*, versus passive anthropogenic transport in *C. ceriferus*, can ultimately lead to the same outcome: persistence and spread under Mediterranean conditions.

### 4.4. Implications and Future Perspectives

The establishment of *Z. tuberculatus* and *C. ceriferus* in Campania reflects a broader trend of repeated incursions of alien insects in Mediterranean regions, where climate warming and plant trade facilitate establishment [48,91]. Comparable situations were described for *Carpophilus truncatus*, reported as being “on the razor’s edge” between outbreak and invasion [10]. Furthermore, the establishment process may be facilitated by the initial scarcity of effective natural enemies, particularly during early invasion stages, in line with the enemy release hypothesis [92]. This hypothesis has been widely invoked to explain the success of both alien invasive species and species expanding their range under climate change. Consistent with this framework, no parasitoid individuals emerged from field-collected fruits in the present study.

Despite the absence of consistent winter records, the recurrent detections of *Z. tuberculatus* over consecutive years suggest that the species is established locally, although its overwintering mechanisms remain uncertain.

A similar difficulty applies to scale insects, which are frequently intercepted on ornamentals. More than half of the exotic species introduced on ornamentals in Italy since 1945 have acclimatised, a trend likely reinforced by climate warming and steady trade in host plants [48]. This highlights the importance of early detection and long-term monitoring, since apparently secondary pests may quickly increase their impact once established [93]. At the same time, the ecological impacts of *Z. tuberculatus* and *C. ceriferus* in Europe remain poorly documented, underlining the need for dedicated impact assessments under Mediterranean conditions.

Genome-wide approaches will be crucial to reveal adaptive pathways invisible to mitochondrial markers, thereby strengthening risk assessments and supporting the design of effective management strategies [15,82].

## 5. Conclusions

This study confirms the establishment of *Z. tuberculatus* in Campania and Lazio and of *C. ceriferus* in Campania, documenting their distribution, host associations, and genetic profiles. *Zaprionus tuberculatus* was found on ten host plants, including three new records, with *D. kaki* emerging as a particularly suitable host, while its ability to reproduce on intact figs underscores the risk for healthy fruits. *Ceroplastes ceriferus* was detected on both fruit and ornamental hosts, with molecular analyses revealing two closely related haplotypes and confirming its acclimatization in southern Italy. Both species show that successful establishment can occur despite reduced genetic variability, provided that ecological flexibility and human-mediated transport favour persistence under favourable climatic conditions. Their contrasting ecological strategies illustrate how different biological traits can nonetheless lead to similar invasion outcomes in Mediterranean environments.

## Figures and Tables

**Table 1:** *Zaprionus tuberculatus*: sampling data including host plant species, locality, coordinates, collection date, number of specimens, and sequencing information (specimen code, mitochondrial haplotype, and Genbank accession number, if available).

Host Plant Species	Locality	Coordinates	Date of Collection	Number of Specimens (Sequenced)	Sequenced Specimen Code	Mt-Haplotype	Accession Number	
							COI	COII
*Citrus reticulata* Blanco	Palma Campania	40°51′08″ N 14°33′14″ E	14 November 2023	7	-	-	-	-
		40°51′05″ N 14°33′17″ E	20 November 2023	10 (2)	AA_725	H1	PX533121	PX644770
					AA_726	H2	PX533137	PX644775
*Citrus sinensis* (L.)	Palma Campania	40°51′52″ N 14°33′07″ E	24 October 2023	3	-	-	-	-
		40°51′53″ N 14°33′18″ E	14 November 2023	1 (1)	AA_724	H2	PX533136	-
		40°51′20″ N 14°33′20″ E		9	-	-	-	-
		40°51′09″ N 14°33′26″ E	20 November 2023	10	-	-	-	-
	Sarno	40°50′30″ N 14°34′11″ E	13 November 2023	9 (1)	AA_736	H1	PX533127	-
*Diospyros kaki*	Palma Campania	40°51′36″ N 14°33′20″ E	20 September 2023	3	-	-	-	-
		40°51′27″ N 14°33′19″ E	18 October 2023	8 (1)	AA_648	H1	PX533117	-
		40°51′18″ N 14°33′24″ E	24 October 2023	1 (1)	AA_635	H2	PX533129	-
		40°51′13″ N 14°33′06″ E		6 (1)	AA_650	H1	PX533119	-
		40°52′06″ N 14°33′08″ E		6 (1)	AA_649	H1	PX533118	-
		40°50′33″ N 14°33′05″ E	31 October 2023	9	-	-	-	-
		40°50′40″ N 14°32′03″ E		7	-	-	-	-
		40°51′09″ N 14°33′26″ E	14 November 2023	4	-	-	-	-
	Vico Equense	40°39′38″ N 14°26′49″ E	14 October 2024	3 (1)	AA_901	H1	PX533125	PX644769
	Roma	42°07′29″ N 12°34′08″ E	5 October 2023	37 (2)	AA_651	H2	PX533131	-
					AA_652	H1	PX533120	-
*Eriobotrya japonica* (Thunb.) Lindl.	Palma Campania	40°51′37″ N 14°33′16″ E	30 May 2024	2 (1)	AA_864	H2	PX533132	-
*Juglans regia* L.	Sarno	40°50′16″ N 14°34′36″ E	20 September 2024	3 (1)	AA_860	H2	PX533133	-
*Malus domestica* (Suckow) Borkh.	Palma Campania	40°52′47″ N 14°32′22″ E	9 January 2024	1 (1)	AA_729	H2	PX533134	-
*Opuntia ficus-indica* (L.) Mill.	Sarno	40°50′30″ N 14°34′11″ E	30 November 2023	3 (3)	AA_728	H1	PX533128	-
					AA_732	H1	PX533122	PX644771
					AA_727	H2	PX533124	PX644776
*Punica granatum* L.	Cisterna di Latina	41°34′9″ N 12°46′39″ E	12 November 2023	28 (2)	AA_730	H1	PX533123	PX644774
					AA731	H2	PX533135	PX644773
*Vitis vinifera*	Sant’ Agnello	40°37′4″ N 14°24′19″ E	22 October 2024	5 (1)	AA_857	H1	PX533126	PX644772
*Ziziphus jujuba* Mill.	Palma Campania	40°51′8″ N 14°33′14″ E	1 October 2024	4 (1)	AA_859	H2	PX533130	-

**Table 2:** *Ceroplastes ceriferus*: sampling data including host plant species, locality, coordinates, collection date, number of specimens, and sequencing information (mitochondrial haplotype and Genbank accession number, if available).

Host Plant Species	Locality	Coordinates	Date of Collection	Number of Specimens (Sequenced)	Sequenced Specimen Code	Mt Haplotype	Accession Number
*Diospyros kaki*	San Giuseppe Vesuviano	40°50′37″ N 14°29′16″ E	4 December 2023	3 (3)	AA658	H1	PX508461
					AA659	H1	PX508462
					AA660	H1	PX508463
	Palma Campania	40°50′52″ N 14°33′28″ E	6 February 2024	3 (1)	BB029	H1	PX508471
		40°51′10″ N 14°32′43″ E	23 February 2024	8 (1)	AA720	H1	PX508470
		40°50′52″ N 14°33′28″ E	14 August 2024	1	-	-	-
	Saviano	40°53′54″ N 14°29′29″ E	5 May 2024	2 (2)	BB032	H1	PX508474
					BB033	H1	PX508475
		40°53′23″ N 14°29′35″ E	7 November 2024	2 (2)	BB035	H1	PX508477
					BB034	H1	PX508476
	San Gennaro Vesuviano	40°51′28″ N 14°30′55″ E	7 November 2024	2 (2)	BB037	H2	PX508479
					BB036	H1	PX508478
*Laurus nobilis* L.	Palma Campania	40°52′46″ N 14°32′59″ E	21 May 2024	27 (3)	AA754	H1	PX508465
					AA752	H1	PX508464
					AA755	H1	PX508466
*Morus* sp.	San Giuseppe Vesuviano	40°49′21″ N 14°31′37″ E	10 October 2024	6 (2)	BB031	H2	PX508473
					BB030	H2	PX508472
*Photinia serratifolia* (Desf.) Kalkman	Ottaviano	40°51′09″ N 14°29′23″ E	2 February 2024	11 (3)	AA717	H1	PX508467
					AA718	H1	PX508468
					AA719	H1	PX508469

**Table 3:** Uncorrected p-distance values based on COI barcoding (top) and COII (bottom) barcoding of *Zaprionus tuberculatus* interspecific distances (±SE) are shown below the diagonal; intraspecific distances (±SE) are in italics along the diagonal, *n.c.:* not calculable. (AFR: Africa; CMR: Cameroon; ITA: Italy; TUR: Turkey).

Country	ITA	TUR	CMR
ITA	*0.16 (±0.112)*		
TUR	0.15 (*±*0.102)	*0.13 (±0.094)*	
CMR	0.20 (*±*0.114)	0.12 (*±*0.071)	*0.1 (±0.103)*
Country	AFR	USA	ITA
AFR	*0.26 (±0.115)*		
USA	1.50 (*±*0.425)	*n.c.*	
ITA	0.63 (*±*0.270)	0.87 (*±*0.334)	*n.c.*

**Table 4:** Uncorrected p-distance values based on COI barcoding of *Ceroplastes ceriferus*. Interspecific distances (±SE) are shown below the diagonal; intraspecific distances (±SE) are in italics along the diagonal. *n.c.*: not calculable. (CHE: Switzerland; CHN: China; ITA: Italy; KOR: South Korea).

Country	ITA	CHN	KOR	CHE
ITA	*0.04 (±0.042)*			
CHN	0.50 (*±*0.154)	*0.76(±0.225)*		
KOR	0.03 (*±*0.028)	0.48 (*±*0.151)	*n.c.*	
CHE	0.14 (*±*0.134)	0.62 (*±*0.218)	0.20 (*±*0.189)	*n.c.*

## Data Availability

All newly generated COI and COII sequences were deposited in GenBank under accession numbers listed in Table 1 and Table 2. All other data supporting the findings of this study are included within the article.

## Supplementary Materials

The following supporting information can be downloaded at: https://www.mdpi.com/article/10.3390/insects17020198/s1, Table S1: COI sequences of *Zaprionus tuberculatus* included in the present study; Table S2: COII sequences of *Zaprionus tuberculatus* included in the present study; Table S3: COI sequences of *Ceroplastes ceriferus* included in the present study.
