# Supplementary material for: Contrasting Invasion Strategies, Convergent Outcomes: Establishment of Zaprionus tuberculatus and Ceroplastes ceriferus in Italy
Source: Insects. 2026 Feb 12;17(2):198. doi: 10.3390/insects17020198 (PMC12941184; doi:10.3390/insects17020198)
Supplement: Supplementary file 1 [file insects-17-00198-s001.zip › insects-4069868-supplementary.pdf]

Contrasting invasion strategies, convergent outcomes: establishment of *Zaprionus tuberculatus* and *Ceroplastes ceriferus* in Italy

Francesco Nugnes[1], Carmela Carbone[1], Fortuna Miele[1], Feliciana Pica[1]*, Sara Pierro[1], Raffaele Sasso[2], Mariagrazia Bodini[1], Umberto Bernardo[1]

[1] Institute for Sustainable Plant Protection - National Research Council (IPSP-CNR), P.le E. Fermi, 1, 80055 Portici, NA, Italy, +3906499324823; francesco.nugnes@cnr.it (F.N.); carmelacarbone@cnr.it (C.C.); fortunamiele@cnr.it (F.M.); sarapierro@cnr.it (S.P.); mariagraziabodini@cnr.it (M.B.); umberto.bernardo@cnr.it (U.B.)
[2] ENEA CR Casaccia, SSPT-AGROS-AGRI4.0, Via Anguillarese, 301, 00123 S. Maria di Galeria - Rome Italy; raffaele.sasso@enea.it
* Correspondence: felicianapica@cnr.it; +39-06-499327826

Supplementary Material

Tab. S1 COI sequences of *Zaprionus tuberculatus* included in the present study

Accession number - Genbank	Country
KX771150	
KX771151	Cameroon
KX771155	
MK509763	
MK509764	
MK509765	Turkey
MK509766	
MK801759	
LN867073	Italy (Apulia)
MW441238	Italy (Sicily)
MW441239	

Tab. S2 COII sequences of *Zaprionus tuberculatus* included in the present study

Accession number - Genbank	Country
EU595373	Zimbawe
EU595372	Uganda
EU595371	Gabon
EU595370	South Africa
EU595369	Republic of the Congo
EU595368	Egypt
EU595367	Niger
EU595366	São Tomé and Príncipe
EU595356	Greece
AF478440	United States of America

Tab. S3 COI sequences of *Ceroplastes ceriferus* included in the present study

**Accession number - Genbank**	**Country**
MZ836057	Switzerland
MK543888	South Korea
KF824021	
JQ795624	
JQ795623	
JQ795609	
JQ795610	
KF824022	
KF824014	
JQ8795656	
JQ795655	
JQ795642	
JQ795614	
JQ795635	
JQ795634	
JQ795636	
JQ795641	China
JQ795692	
JQ795691	
JQ795684	
JQ795683	
JQ795662	
JQ795661	
JQ795612	
JQ795613	
JQ795615	
JQ795622	
JQ795640	
JQ795657	
JQ795611	
MW450919	
